# Large-Scale Off-Target Identification Using Fast and Accurate Dual Regularized One-Class Collaborative Filtering and Its Application to Drug Repurposing

**DOI:** 10.1371/journal.pcbi.1005135

**Published:** 2016-10-07

**Authors:** Hansaim Lim, Aleksandar Poleksic, Yuan Yao, Hanghang Tong, Di He, Luke Zhuang, Patrick Meng, Lei Xie

**Affiliations:** 1 The Graduate Center, The City University of New York, New York, New York, United States; 2 Department of Computer Science, University of Northern Iowa, Cedar Falls, Iowa, United States; 3 Department of Computer Science and Technology, Nanjing University, Nanjing, Jiangsu, China; 4 School of Computing, Informatics and Decision Systems Engineering, Arizona State University, Tempe, Arizona, United States; 5 Academy for Information Technology, Union County Vocational-Technical Schools, Scotch Plains, New Jersey, United States; 6 High Technology High School, Lincroft, New Jersey, United States; 7 Department of Computer Science, Hunter College, The City University of New York, New York, New York, United States; Icahn School of Medicine at Mount Sinai, UNITED STATES

## Abstract

Target-based screening is one of the major approaches in drug discovery. Besides the intended target, unexpected drug off-target interactions often occur, and many of them have not been recognized and characterized. The off-target interactions can be responsible for either therapeutic or side effects. Thus, identifying the genome-wide off-targets of lead compounds or existing drugs will be critical for designing effective and safe drugs, and providing new opportunities for drug repurposing. Although many computational methods have been developed to predict drug-target interactions, they are either less accurate than the one that we are proposing here or computationally too intensive, thereby limiting their capability for large-scale off-target identification. In addition, the performances of most machine learning based algorithms have been mainly evaluated to predict off-target interactions in the same gene family for hundreds of chemicals. It is not clear how these algorithms perform in terms of detecting off-targets across gene families on a proteome scale. Here, we are presenting a fast and accurate off-target prediction method, REMAP, which is based on a dual regularized one-class collaborative filtering algorithm, to explore continuous chemical space, protein space, and their interactome on a large scale. When tested in a reliable, extensive, and cross-gene family benchmark, REMAP outperforms the state-of-the-art methods. Furthermore, REMAP is highly scalable. It can screen a dataset of 200 thousands chemicals against 20 thousands proteins within 2 hours. Using the reconstructed genome-wide target profile as the fingerprint of a chemical compound, we predicted that seven FDA-approved drugs can be repurposed as novel anti-cancer therapies. The anti-cancer activity of six of them is supported by experimental evidences. Thus, REMAP is a valuable addition to the existing *in silico* toolbox for drug target identification, drug repurposing, phenotypic screening, and side effect prediction. The software and benchmark are available at https://github.com/hansaimlim/REMAP.

## Author Summary

High-throughput techniques have generated vast amounts of diverse omics and phenotypic data. However, these sets of data have not yet been fully explored to improve the effectiveness and efficiency of drug discovery, a process which has traditionally adopted a one-drug-one-gene paradigm. Consequently, the cost of bringing a drug to market is astounding and the failure rate is daunting. The failure of the target-based drug discovery is in large part due to the fact that a drug rarely interacts only with its intended receptor, but also generally binds to other receptors. To rationally design potent and safe therapeutics, we need to identify all the possible cellular proteins interacting with a drug in an organism. Existing experimental techniques are not sufficient to address this problem, and will benefit from computational modeling. However, it is a daunting task to reliably screen millions of chemicals against hundreds of thousands of proteins. Here, we introduce a fast and accurate method REMAP for large-scale predictions of drug-target interactions. REMAP outperforms state-of-the-art algorithms in terms of both speed and accuracy, and has been successfully applied to drug repurposing. Thus, REMAP may have broad applications in drug discovery.

## Introduction

Conventional one-drug-one-gene drug discovery and drug development is a time-consuming and expensive process. It suffers from high attrition rate and possible unexpected post-market withdrawal [1]. It has been recognized that a drug rarely only binds to its intended target, and off-target interactions (i.e. interactions between the drug and unintended targets) are common [2]. The off-target interaction may lead to adverse drug reactions (ADRs) [3], as demonstrated by the deadly side effect of a Fatty Acid Amide Hydrolase (FAAH) inhibitor in a recent clinical trial [4]. On the other hand, the off-target interaction may be therapeutically useful, thus providing opportunities for drug repurposing and polypharmacology [2]. Therefore, identifying off-target interactions is an important step in drug discovery and development in order to reduce the drug attrition rate and to accelerate the drug discovery and development process, and ultimately to make safer and more affordable drugs.

Many efforts have been devoted to developing statistical machine learning methods for the prediction of unknown drug-target associations by screening large chemical and protein data sets [5]. One of the fundamental assumptions in applying statistical machine learning methods to drug-target interaction prediction is that similar chemicals bind to similar protein targets, and vice versa. Based on this similarity principle, both semi-supervised and supervised machine learning techniques have been applied. The semi-supervised learning methods either build statistical models for the *k* nearest neighbors (*k-*NN) of the query compound with similar compounds in the database (e.g. Parzen-Rosenblatt Window (PRW) [6] and Set Ensemble Analysis (SEA) [7] are examples). Although a large number of 2D and 3D fingerprint representations of chemical structures have been developed, chemical structure similarity that is measured by Tanimoto coefficient (TC) or other similarity metrics of fingerprints is not continuously correlated with the binding activity. Activity cliff exists in the chemical space, where a small modification of a chemical structure can lead to a dramatic change in binding activity [8]. Thus, the chemical structural similarity alone is not sufficient to capture genome-wide target binding profile, as protein-chemical interaction is determined by both protein structures and chemical structures. New deep learning techniques that can learn non-linear, hierarchical relationships may provide new solutions for representing chemical space [9–12]. However, few work has been done to incorporate protein relationships into the deep learning framework. It remains to be seen whether the deep learning is applicable to genome-wide target prediction.

A number of techniques such as Gaussian Interaction Profile (GIP), Weighted Nearest Neighbor (WNN), Regularized Least Squares (RLS) classifier [13, 14], and matrix factorization [15–17] have been developed to integrate chemical and genomic space. Among them, Neighborhood Regularized Logistic Matrix Factorization (NRLMF) [17] and Kernelized Bayesian Matrix Factorization (KBMF) [16] are two of the most successful methods. However, several drawbacks in these algorithms hinder their applications in genome-wide off-target predictions. First, several algorithms with high performance such as KBMF are extremely time and memory-consuming. Second, these algorithms depend on a supervised learning framework that requires negative cases. While publicly available biological and/or chemical databases (e.g. ZINC [18], ChEMBL [19], DrugBank [20], PubChem [21], and UniProt [22]) have enabled large-scale screening of drug-target associations, the known chemical-protein associations are sparse, and the number of reported negative cases (i.e. chemical-protein pairs not associated) is too small to optimally train a prediction algorithm [23]. Using randomly generated negative cases will adversely impact the performance of these algorithms, and algorithmically derived negative cases are often based on unrealistic assumptions [23]. Finally, these algorithms have been mainly evaluated for the prediction of off-targets within the same gene family (e.g. GPCR) using a small benchmark with hundreds of drugs and targets. Their performances in predicting off-target across gene families on a large scale are uncertain. Indeed, drug cross-reactivity often occurs across fold spaces [2]. Thus, the development of *in silico* prediction methods that are fast as well as accurate enough to explore the available data is urgent.

Here, we make several contributions to address the aforementioned problems. First, we present an efficient method, REMAP, which formulates the off-target predictions as a dual-regularized One Class Collaborative Filtering (OCCF) problem. Thus, negative data are not needed for the training, but can be used if available. Secondly, REMAP is highly scalable with promising accuracy, thus can be applied to large-scale off-target predictions. Thirdly, we introduce a new benchmark set to evaluate the performance of drug-target interactions across gene families. Finally, we apply REMAP to repurposing existing drugs for new diseases. We identified seven drugs that have anti-cancer activity. Six of them are supported by experimental evidence.

## Materials and Methods

### Problem formulation

The problem we try to solve here is to predict how likely it is that a chemical interacts with a target protein, using a chemical-protein association network, chemical-chemical similarity, and protein-protein similarity information. We start by preparing a bipartite network for chemical-protein associations as a sparse *n* × *m* matrix *R*, where *n* is the number of chemicals and *m* is the number of proteins. *R*_*i*,*j*_ = 1 if the *i*^*th*^ chemical is associated with the *j*^*th*^ protein, and *R*_*i*,*j*_ = 0, otherwise. The chemical-chemical similarity scores are in an *n* × *n* square matrix *C*, with *C*_*i*,*j*_ representing the chemical-chemical similarity score between the *i*^*th*^ and *j*^*th*^ chemicals (0 ≤ *C*_*i*,*j*_ ≤ 1) for total *n* chemicals. The protein-protein similarity scores are in the same format for total *m* proteins (0 ≤ *T*_*i*,*j*_ ≤ 1). We consider this problem an analog of user-item preferences such that users and items represent chemicals and proteins, respectively. Therefore, the problem is to provide an *n* × *m* matrix *P* in which *P*_*i*,*j*_ is the prediction score for the interaction between the *i*^*th*^ chemical and the *j*^*th*^ protein.

### Overview of off-target prediction method REMAP

Our prediction method REMAP is based on a one-class collaborative filtering algorithm that recommends the users’ preferences to the listed items [24]. It assumes that similar users will prefer similar items, unobserved associations are not necessarily negative, and user-item preferences can be analogous to drug-target associations. Assuming that a fairly low number of factors (i.e. smaller number of features than the number of total chemicals or protein targets) may capture the characteristics determining the chemical-protein associations, two low-rank matrices, *U* (chemical side) and *V* (protein side), were approximated such that $\sum_{i}^{n}\sum_{j}^{m}\left\{R-\left(U\cdot V^{T}\right)\right\}$ is minimized where *R* is the matrix for known chemical-protein associations and *V*^*T*^ is the transposition of the protein side low-rank matrix *V*. The two low rank matrices, *U*_*n×r*_ and *V*_*m×r*_ are obtained by iteratively minimizing the objective function,

$${\text{min}}_{U,V\geq 0}\sum_{\left(i,j\right)}p_{wt}{\left(R_{\left(i,j\right)}+p_{imp}-U_{\left(i,:\right)}\cdot V_{\left(j,:\right)}^{T}\right)}^{2}+p_{reg}\left({\left\|U\right\|}^{2}+{\left\|V\right\|}^{2}\right)+p_{chem}tr\left(U^{T}\left(D_{C}-C\right)U\right)+p_{prot}tr\left(V^{T}\left(D_{T}-T\right)V\right)$$ (1)

**Fig 1:**
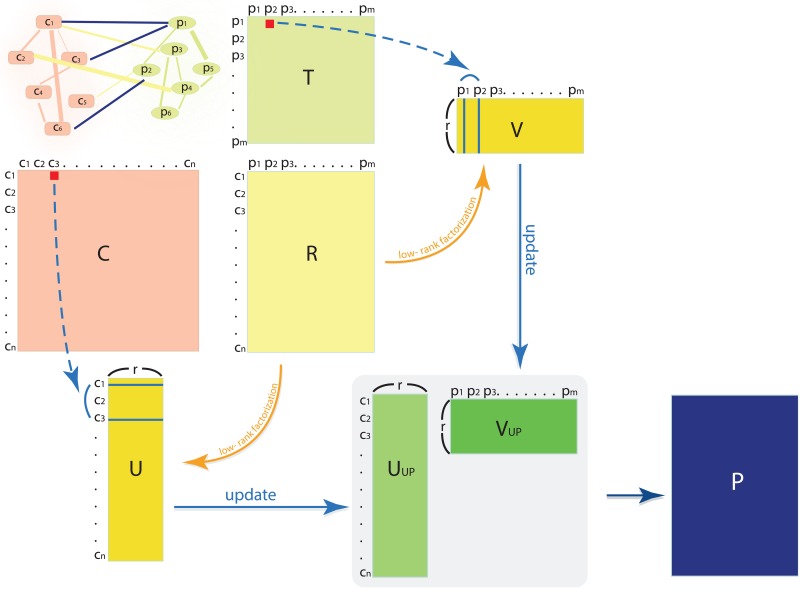
The overall process of REMAP. The rectangular boxes with capitalized symbols are matrices, and the smaller boxes and ovals are chemicals and proteins, respectively, in the simplified network representation (top-left corner). Solid lines within the network represent connectivity (edges), and the arrows represent mathematical processes. Red squares represent single similarity values, and blue bars in U and V represent row and column vectors. Lower-case c and p represents chemicals and proteins, respectively. The letter symbols are annotated in Table 1.

All symbols used in the paper are summarized in Table 1, and the overall process of REMAP is in Fig 1. Here, *p*_*wt*_ is the penalty weight on the observed and unobserved associations which indicate the reliability of the assigned probability of true association, *p*_*imp*_ is the imputed value (i.e. the probability of unobserved associations as real associations), *p*_*reg*_ is the regularization parameter to prevent overfitting, *p*_*chem*_ is the importance parameter for chemical-chemical similarity, *p*_*prot*_ is the importance parameter for protein-protein similarity, and *tr*(A) is the trace of matrix A (Table 1). In this study, we use global weight and imputation. However, the weight and imputation values may be determined by *a priori* knowledge or from the prediction of other machine learning algorithms (i.e. *p*_*wt*_ and *p*_*imp*_ can be matrices with the same dimension as the matrix *R*). The raw predicted score for the *i*^*th*^ chemical to bind the *j*^*th*^ protein can be calculated by $P_{\left(i,j\right)}=U_{UP\left(i,:\right)}\cdot V_{UP\left(j,:\right)}^{T}$. The raw scores were adjusted based on the ratio of observed positive and negative cases when the negative data are available (explained in the prediction score adjustment section). Also, the matrix *U*_*n×r*_ is referred to as a low-rank drug profile since its *i*^*th*^ row represents the *i*^*th*^ drug’s behavior in the drug-target interaction network as well as drug-drug similarity spaces compressed to *r* number of features. The REMAP code was originally written in Matlab and modified for drug-target predictions.

**Table 1:** The symbols and the descriptions for numerical calculations

Symbol	Definition and Description
*R*	The adjacency matrix of the known drug-target associations
*C*, *T*	The chemical-chemical and the target-target similarity matrices
$C_{\;(c_{1},c_{2})}$	The chemical-chemical similarity score for the chemicals *c*_1_ and *c*_2_
$d_{Tani\;(c_{1},c_{2})}$	The Tanimoto dissimilarity coefficient for the chemicals *c*_1_ and *c*_2_
*T*_(*p*1,*p*2)_	The target-target similarity score for the query protein *p*_1_ and the target protein *p*_2_
*d*_*bit*(*p*1,*p*2)_	The bit score for the query protein *p*_1_ and the target protein *p*_2_
*D*_*C*_, *D*_*T*_	The degree matrices of *C* and *T*, respectively
*U*, *V*	The chemical-side and the target-side low-rank approximation matrices
*R*_(*i*,*j*)_	The element of *R* at its *i*^*th*^ row and *j*^*th*^ column
*R*_(*i*,;)_	The *i*^*th*^ row of *R*
*R*_(;,*j*)_	The *j*^*th*^ column of *R*
*R*^*T*^	The transpose matrix of *R*
*tr*(*R*)	The trace of *R*
*p*_*wt*_	The penalty weight on observed and unobserved associations which indicate the reliability of assigned probability of true association
*p*_*imp*_	The imputed value (i.e. the probability of unobserved associations as real associations
*p*_*reg*_	The regularization parameter to prevent overfitting
*p*_*chem*_	The importance parameter for chemical-chemical similarity
*p*_*prot*_	The importance parameter for protein-protein similarity
*r*	The rank of the low-rank approximation matrices
*p*_*iter*_	The number of maximum iterations to minimize the objective function
*p*_(*i*,*j*)_	The raw prediction score by REMAP for the *i*^*th*^ chemical and the *j*^*th*^ protein

### Chemical-chemical similarity

Chemical-chemical similarity scores are one of the required inputs of REMAP. Although there are a number of metrics developed for chemical-chemical similarity, a recent study showed that Tanimoto coefficient-based similarity is highly efficient for fingerprint-based similarity measurement [25]. The fingerprint of choice in this study is the Extended Connectivity Fingerprint (ECFP), which has been successfully applied to chemical structure-based target prediction method, PRW [6]. Thus, it allows for a fair comparison of REMAP with PRW. It is interesting to compare the different fingerprints in the future study.

To calculate a similarity score between two chemicals, c_1_ and c_2_, the Tanimoto dissimilarity coefficient $d_{Tani\;(c_{1},c_{2})}$ was obtained using JChem with the Tanimoto metric for the ECFP descriptor type using the command in the Unix environment, “*ChemAxon/JChem/bin/screenmd target_smi query_smi -k ECFP -g -c -M Tanimoto*” [26]. The chemical-chemical similarity score, $C_{\;(c_{1},c_{2})}$ is defined as $C_{\;(c_{1},c_{2})}\;=\;1-d_{Tani\;(c_{1},c_{2})}$. Briefly, two chemicals have a higher similarity score if they have more of the same chemical moieties (e.g. functional groups) at more similar relative positions. Chemical similarity scores below 0.5 were treated as noise and set to 0.

### Protein-protein similarity

Protein-protein similarity scores are also one of the required inputs for REMAP. The similarity between two proteins was calculated based on their sequence similarity using NCBI BLAST [27] with an e-value threshold of 1 × 10^−5^ and its default options (e.g. 11 for gap open penalty and 1 for its extension, BLOSUM62 for the scoring matrix, and so on). Based on our 10-fold cross validation (see below), e-value thresholds from 1 to 1 × 10^−20^ did not significantly affect the performance (S1 Fig). Therefore, we decided to use a moderately stringent threshold (BLAST default is 1 × 10^−3^). A similarity score for query protein *p*_1_ to target protein *p*_2_ was calculated by the ratio of a bit score for the pair compared to the bit score of a self-query. To be specific, for the query protein *p*_1_ to the target protein *p*_2_, protein-protein the similarity score was defined such that *T*_(*p*1,*p*2)_ = *d*_*bit*(*p*1,*p*2)_/*d*_*bit*(*p*1,*p*1)_.

### Benchmark test and data preparation

For benchmark tests, ZINC data was filtered by IC_50_ ≤ 10 μM, which yielded 31,735 unique chemical-protein associations for 12,384 chemicals and 3,500 proteins (ZINC dataset [18]). Targets that are protein complexes or cell-based tests were excluded. Proteins whose primary sequence is unavailable were also excluded. Protein sequences were obtained from UniProt [22], and the whole protein sequences were used to calculate protein-protein similarity scores.

To assess the predictive power of our algorithm, we performed a 10-fold cross validation on the ZINC dataset described above. We set the parameters as follows: *p*_*wt*_ = *p*_*imp*_ = *p*_*reg*_ = 0.1, *r* = 300, *p*_*chem*_ = 0.75, *p*_*prot*_ = 0.1, and *p*_*iter*_ = 400. The optimized values determined by the 10-fold cross validation of benchmark are shown in S2 Fig. It is noted that the best performance is achieved when *p*_*chem*_ = 0.25 and *p*_*prot*_ = 0.25. To further evaluate REMAP, we compared its performance on the ZINC dataset with several methods: a chemical similarity-based method (PRW [6]), the best performed matrix factorization methods so far (NRLMF [17] and KBMF with twin kernels (KBMF2K) [16]), combination of WNN and GIP (WNNGIP [14]), and another type of matrix factorization method (Collaborative Matrix Factorization (CMF) [15]) for different types of chemicals and proteins.

To obtain a detailed view of the performance of the methods, we divided the ZINC dataset into 3 categories with 2 subcategories for each, based on the connectivity of known chemical-protein associations and the degree of uniqueness of the chemicals. First, all the chemicals in the dataset were classified into the chemicals having only one known target (NT1), two known targets (NT2), or three or more known targets (NT3). Then, for the chemicals in each category, they were further divided based on either the number of known chemicals (ligands) the target proteins are associated with (number of ligands in increments of 5) or the maximum chemical-chemical similarity score for the chemical in the dataset (the similarity score range increment is 0.1). The label used in this paper for the dataset are NT*a*L*b*, or NT*a*MaxTc*d*, where ‘NT’ stands for the Number of known Target, ‘L’ for the number of known Ligand, and ‘Tc’ for the maximum (Tanimoto coefficient-based) chemical-chemical similarity score for the given chemical in the dataset, with NT = *a*, *b* ≤ L ≤ *b* +4, and *d* − 0.1 < Tc ≤ *d*. For instance, NT2L1 is the data set label for chemicals having two known targets and proteins having 1 to 5 ligands in the dataset, and NT1Tc0.9 is for chemicals with the most similar chemicals between 0.8 and 0.9 of similarity scores and having one known target. Chemicals having more than three known targets are included in the NT3 class, and proteins having more than twenty-one known ligands were included in L21 (not limited to 25). The categories of the ZINC dataset were then used to evaluate the performance of off-target prediction, and their labels mean the number of known ligands (L) or the maximum structural similarity (Tc) with their corresponding ranges. For example, ‘L21more’ stands for the dataset for proteins having 21 or more known targets, and ‘Tc0.9to1.0’ stands for maximum structural similarity greater than 0.9 and up to 1.0 (Tc0.5to0.6 is inclusive of 0.5). Note that NT1 is equivalent to chemicals without any known target when they are tested for cross validation. Therefore, performances on NT1 datasets reflect the ability to address the *cold start* problem. In other words, when one known drug-target association is intentionally hidden for the chemicals in the NT1 dataset, the tested chemicals will not have any known target in the training data, and they are less likely to be given a good recommendation of targets. This is analogous to the *new user or new item* problem reviewed by Su et al. [28].

### Measuring prediction accuracy of REMAP by TPR *vs*. cutoff rank

A typical measure of prediction performance is the Receiver Operating Characteristic (ROC) curve by which one can assess the reliability of the positively predicted results. However, it is difficult to apply the ROC curve on our chemical-protein association datasets since the vast majority of the chemical-protein pairs have not been tested, and thus it is unclear whether the missing entries are actually unassociated or just not yet observed.

In order to assess how reliable the positively predicted results from REMAP are, we needed to define a performance measurement that is analogous to ROC curve but not dependent on the true negatives. Our primary measure of performance is the true positive rate ($\frac{\sum True\;Positives}{\sum Condition\;Positives};$ Recall or Recovery) at the top 1% of predictions for each chemical. To be specific, the top 1% of predictions includes up to the 35^th^-ranked predicted target protein for a chemical for our datasets (3,500 possible target proteins for each chemical). Thus, for instance, a TPR of 0.965 at the 35^th^ cutoff rank (top 1%) means that 96.5% of the total tested positive pairs were ranked 35^th^ or better for the tested chemicals.

### Scalability of REMAP as a matrix factorization algorithm

In order to assess the speed of REMAP for practical uses, we measured its running time by varying the rank parameter or the size of dataset. On the ZINC dataset (12,384 chemicals and 3,500 proteins), up to *r* = 2,000 was tested, and at fixed *r* = 200, dataset sizes up to 200,000 chemicals and 20,000 proteins were tested. The number of iterations (*p*_*iter*_) was fixed to 400. A single node of CPU with 2.88 GB of memory in the City University of New York High Performance Computing Center (CUNY HPCC) was used for REMAP running time tests. We also compared the running times of different matrix factorization methods with ours. Due to the large time complexity and memory requirement for other algorithms, a multi-core node with up to 700 GB of shared memory system in CUNY HPCC was used for them on the ZINC dataset.

### Genome-wide chemical-protein associations

Chemical-protein associations were obtained from the ZINC [18], ChEMBL [19] and DrugBank [20] databases. To obtain reliable chemical-protein association pairs, binding assays records with IC_50_ information were extracted from the databases, and the cutoff IC_50_ value of 10 μM was used where applicable. Two chemicals were considered the same if their InChI Keys are identical, and two proteins were considered so if their UniProt Accessions are identical. For records with IC_50_ in μg/L (found in ChEMBL), the full molecular weights of the compounds listed on ChEMBL were used to convert μg/L to μM. Chemical-protein pairs were considered associated if IC_50_≤10 μM (active pairs), unassociated if IC_50_>10 μM (inactive pairs), ambiguous if records exist in both ranges (ambiguous pairs), and unobserved otherwise (unknown pairs). A total of 198,712 unique chemicals and 3,549 unique target proteins were obtained from the combination of ChEMBL and ZINC with 228,725 unique chemical-protein active pairs, 76,643 inactive pairs, and 4,068 ambiguous pairs. Of the 198,712 chemicals, 722 were found to be FDA-approved drugs. Furthermore, drug-target relationships were extracted from the DrugBank and integrated into the ZINC_ChEMBL dataset above. A total of 199,338 unique chemicals and 6,277 unique proteins were obtained from the combination of ZINC, ChEMBL, and DrugBank with 233,378 unique chemical-protein active pairs.

### Drug-target interaction profile analysis for drug repurposing

Since REMAP showed promising performances on predicting off-targets for chemicals with at least one known target, it is possible to use REMAP to suggest new purposes for some FDA approved drugs. As the matrix product of *U*_*UP*_ (chemical-side low-rank matrix) and *V*_*UP*_ (protein side low-rank matrix) is the predicted drug-target interaction matrix *P*, the *i*^*th*^ row of *U*_*UP*_ contains the target interaction profile for the *i*^*th*^ drug. Therefore, we analyzed the drug-drug similarities based on the low-rank matrix *U*_*UP*_. We ran REMAP with the data combination of three databases explained above, with the parameters used in the benchmark evaluations. Then, we calculated drug-drug cosine similarities based on the matrix *U*_*UP*_. For each row of *U*_*UP*_ for FDA approved drugs, the cosine similarity of drug c_1_ and drug c_2_ can be calculated by, $S_{cos,(c_{1},c_{2})}\;=\;\frac{\vec{U_{c_{1}}}∙\vec{U_{c_{2}}}}{\sqrt{\left|U_{c_{1}}\right|}\sqrt{\left|U_{c_{2}}\right|}}$. To search for possibly undiscovered uses of the drugs, we focused on drugs that are found to have high cosine similarity but low Tanimoto similarity (< 0.5). Markov Cluster (MCL) Algorithm [29, 30] was used to cluster drugs based on their cosine similarity of a low-rank target profile. Drug-disease associations were obtained from the Comparative Toxicogenomics Database (CTD) [31].

### Prediction score adjustment

**Fig 2:**
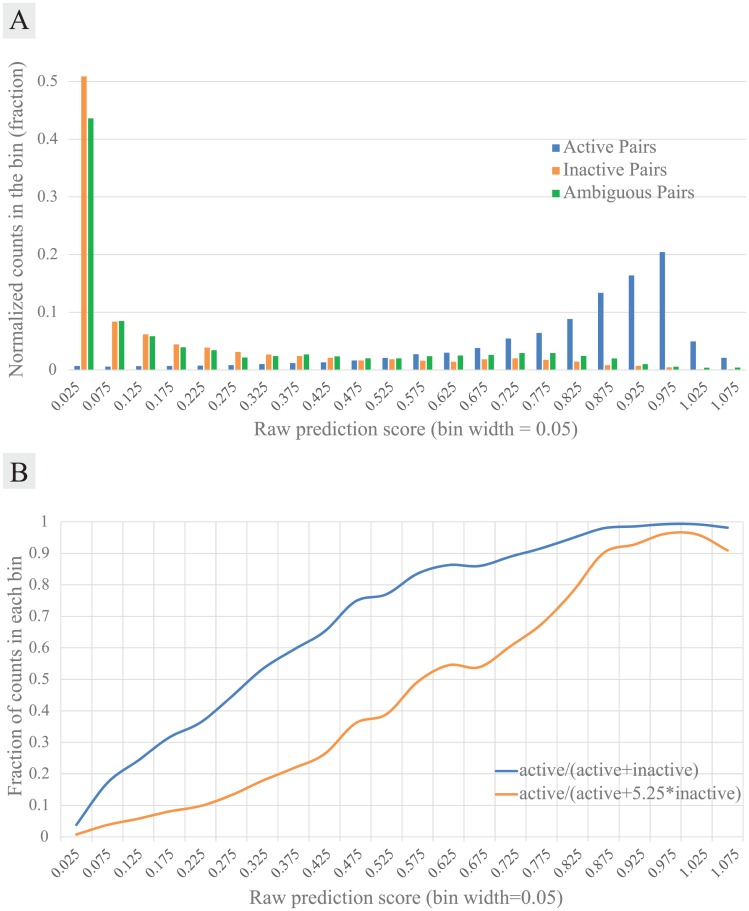
**(A) REMAP score distributions for active (blue), inactive (orange), and ambiguous (green) pairs.** For each bin of raw prediction scores (x-axis, bin width = 0.05), the number of pairs found in the bin was divided by the total of the type of data (total numbers in the plot). Raw prediction scores over 1.10 were regarded as outliers and not included in the figure. Active pairs were obtained from the ZINC and the ChEMBL databases, and inactive, and ambiguous pairs were obtained from the ChEMBL database. **(B)** Adjusted scores for each bin of raw prediction scores (x-axis, same bin width as A). Adjustment by the counts only (blue) and adjustment with weighted counts (orange). A weight of 5.25 was given for the counts of inactive pairs as explained in the prediction score adjustment section.

The raw prediction score ($P_{\left(i,j\right)}\;=\;U_{UP(i,:)}{∙V}_{UP(j,:)}^{T}$) can be adjusted to better reflect the real data as well as to statistically discriminate the positive and negative predictions. We used the active, inactive and ambiguous pairs obtained from the ChEMBL database to adjust the score. REMAP prediction on the ZINC_ChEMBL dataset showed a clear division between the active and inactive pairs, suggesting that predictions scored around 1.0 are highly likely to be positive (Fig 2A). As mentioned above, however, there is a large difference between the number of active and inactive pairs, which is not likely to reflect the ratio of the actual positive and negative chemical-protein pairs. Greater accuracy is expected by adjusting the prediction scores to reflect such a positive/negative ratio. To estimate the ratio, we first normalized the counts in each bin in the histogram (Fig 2A) and calculated the weights that minimize the sum of error, *E*_*sum*_. *E*_*sum*_(*w*_1_) = Σ_*i*_[*A*_*i*_ − {*w*_1_*p*_*i*_ + (1 − *w*_1_)*N*_*i*_}]^2^, where *w*_1_ and w_2_ are the weights on active and inactive pairs, respectively (*w*_1_ + *w*_2_ = 1.0), and *A*_*i*_, *p*_*i*_ and *N*_*i*_ are the normalized counts in *i*^*th*^ bin of ambiguous, active and inactive pairs, respectively. The optimum adjustment weights were approximately *w*_1_ = 0.16, *w*_2_ = 0.84 (Fig 2B). This implies that approximately 16% of total observations are positive. Since the ratio of negative/positive is about 5.25 $\left(\frac{w_{2}}{w_{1}}\;=\;5.25\right)$, we increased the number of observations for inactive pairs in each bin by 5.25 times and rounded down. The adjusted prediction score for each bin (*B*_*i*_) was calculated using the increased negative counts.

$$B_{i}=\frac{\sum number\;of\;positive\;observations}{\sum number\;of\;positive\;observations+5.25\cdot \sum number\;of\;negative\;observations}$$ (2)

It is noted that the prediction score adjustment was not used in the benchmark study, where no negative data were used.

### Graphic analysis

Drug-drug clustered network was visualized using Cytoscape [32].

## Results

### REMAP is highly effective in predicting off-targets even for novel chemicals

We evaluated the performances of algorithms for chemicals having one, two, or more than three known targets with varying maximum chemical-chemical similarity ranges or with proteins having a certain number of known ligands (dataset prepared as explained in the methods and materials section). In general, the performances of both algorithms improve as the number of known ligands per protein or the maximum chemical-chemical similarity value increases.

**Fig 3:**
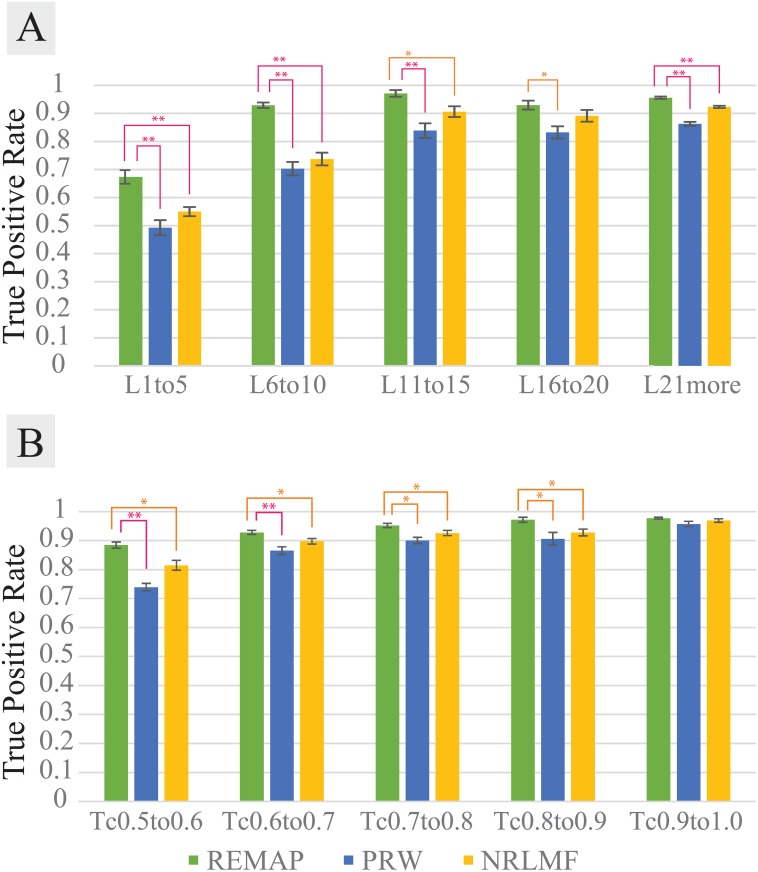
Performance comparison for REMAP (green), PRW (blue), and NRLMF (orange). NT2 (2 known targets per chemical) datasets used for varying number of ligands (A) and chemical structural similarity (B). Performance measurement explained in the measuring prediction accuracy of REMAP by TPR vs. cutoff rank section. **(A)** Performance comparison on the datasets with varying number of ligands per protein. For example, the x-axis of L11to15 means that the proteins of interest have between 11 and 15 known chemicals to bind. **(B)** Performance comparison on the datasets with the ranges of chemical structural similarity of the tested chemicals to the trained chemicals. For instance, the x-axis of Tc0.6to0.7 means that for the tested chemicals, at least one trained chemical was found such that $0.6<C_{\left(c_{1},c_{2}\right)}\leq 0.7$ and no trained chemical was found in greater similarity than 0.7. All TPR values are based on 10-fold cross validation. Error bars represents s.e.m. Asterisks represents statistical significance based one t-test of the 10 TPR values (* for p < 0.05, ** for p < 0.001).

**Fig 4:**
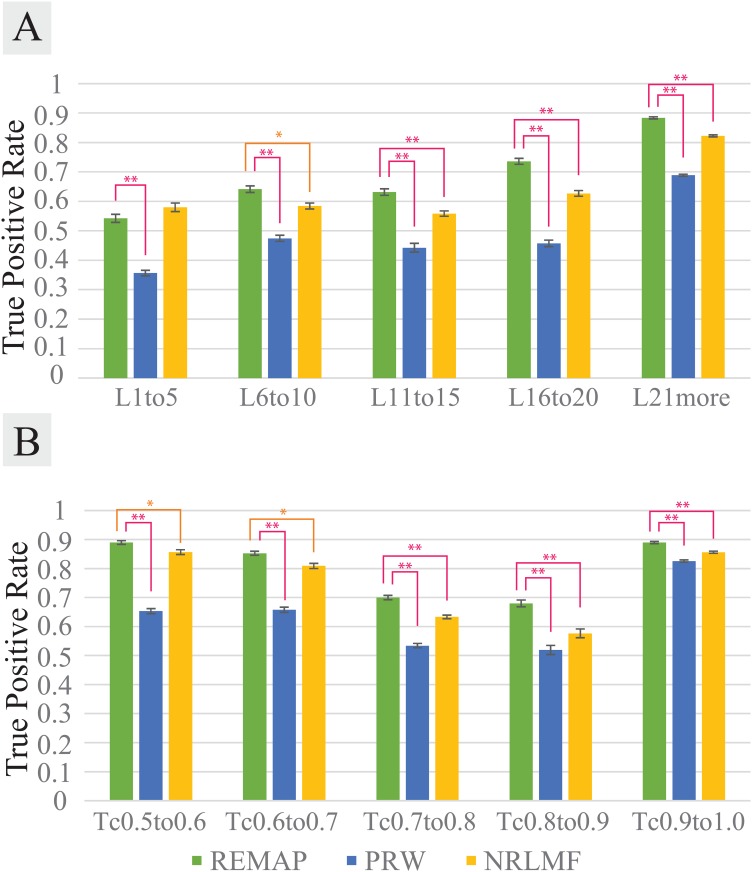
Performance comparison for REMAP (green), PRW (blue), and NRLMF (orange). NT3 (3 or more known targets per chemical) datasets used for varying number of ligands (A) and chemical structural similarity (B). Performance measurement explained in the measuring prediction accuracy of REMAP by TPR vs. cutoff rank section. **(A)** Performance comparison on the datasets with varying number of ligands per protein. For example, the x-axis of L21more means that the proteins of interest have 21 or more known chemicals to bind. **(B)** Performance comparison on the datasets with the ranges of chemical structural similarity of the tested chemicals to the trained chemicals. For instance, the x-axis of Tc0.5to0.6 means that for the tested chemicals, at least one trained chemical was found such that $0.5\leq C_{\left(c_{1},c_{2}\right)}\leq 0.6$ and no trained chemical was found in greater similarity than 0.6. All TPR values are based on 10-fold cross validation. Error bars represents s.e.m. Asterisks represents statistical significance based one t-test of the 10 TPR values (* for p < 0.05, ** for p < 0.001).

It was noticeable that REMAP performed significantly better than PRW when there was at least one known target for a chemical whose targets are predicted (Figs 3 and 4). REMAP showed greater than 90% recovery at the top 1% when the tested chemicals have at least one known target. All algorithms are sensitive to the number of ligands per target. The more ligands, the higher accuracy. While PRW also reached reasonably high recovery for some categories (e.g. more than 11 known ligands per proteins, or $C_{\left(c_{1},c_{2}\right)}>0.6$ of the most similar trained chemicals), REMAP showed that it is reliable for testing chemicals without high similarity to the trained chemicals (Figs 3B and 4B). In other words, REMAP is applicable to chemicals that are structurally distant to the chemicals already in the dataset. Except where the target proteins have 1 to 5 known ligands, REMAP performed best among the three algorithms in all cases with at least one known target for the tested chemicals (Figs 3 and 4). In the most of cases, the differences in the performance between REMAP and other two algorithms are statistically significant. Therefore, in practice, REMAP can predict potential drug targets for chemicals with at least one known target as training data, even when the chemicals are structurally dissimilar to the training chemicals. With the optimized parameters (see below), ROC-like curves shows the general trend of performances of the three algorithms up to the top 10% of predictions (S3 and S4 Figs).

As shown in Figs 3 and 4, REMAP outperforms the state-of-the-art NRLFM algorithm in most of the tested cases. As NRLMF is sensitive to the rank parameter, we carried out optimizations to determine optimal rank and iterations for NRLMF (S5 Fig). The optimal rank and iterations used in the evaluation were 100 and 300, respectively. Moreover, in the current implementation, REMAP is approximately 10 times faster and uses 50% less memory than NRLMF. Consistent with the results by Liu et al. [17], the accuracies of NRLFM are significantly higher than KBMF2K, CMF, and WNNGIP in all of ZINC benchmarks. Overall, REMAP is one of the best-performing methods for the genome-wide off-target predictions.

### Chemical-chemical similarity based on Tanimoto coefficient significantly helps REMAP’s performance, while protein-protein similarity information contains significant noise

**Fig 5:**
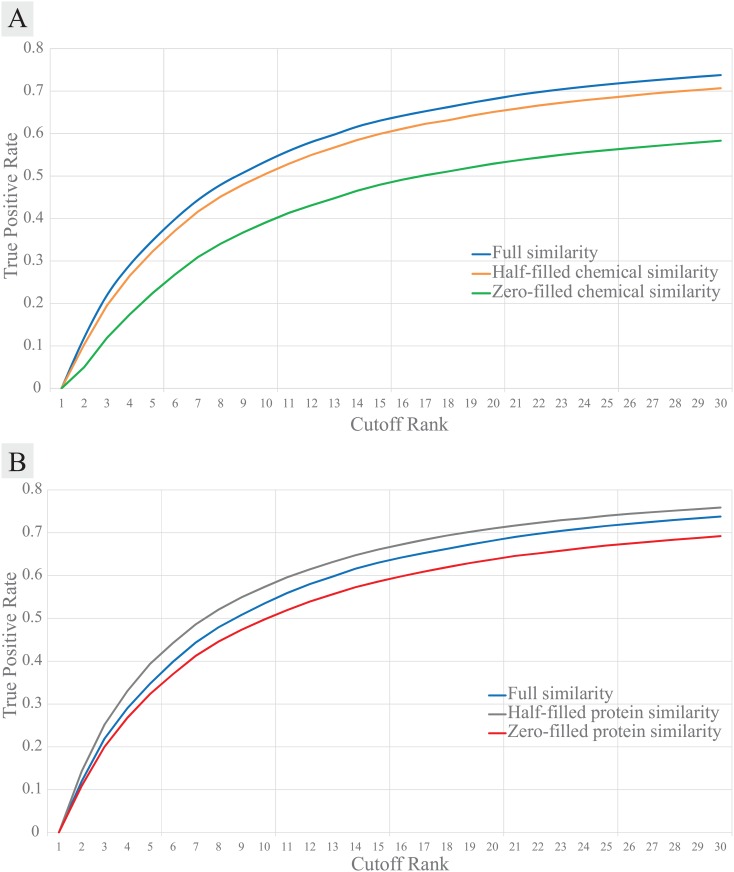
Performance of REMAP according to the amount of the chemical-chemical or the protein-protein similarity information used for its 10-fold cross validation on the ZINC dataset. **(A)** True Positive Rate at the given cutoff rank. All available chemical and protein similarity information included (blue), a half of chemical-chemical similarity was ignored (orange), and the entire chemical-chemical similarity was ignored (green). **(B)** The blue line is the same as A. A half of protein-protein similarity matrix was ignored (gray), and the entire protein-protein similarity was ignored (red).

To test whether the chemical-chemical similarity matrix helps prediction, we performed 10-fold cross validation on the ZINC dataset with the contents of the chemical-chemical or the protein-protein similarity matrix controlled. In other words, about half of the non-zero chemical-chemical similarity scores were randomly chosen and removed (set to 0) for the “half-filled chemical similarity” matrix, and all entries are set to 0 for the “zero-filled chemical similarity” matrix. The predictive power of REMAP showed noticeable improvement when all available chemical-chemical similarity pairs were used, compared to the half-filled or the zero-filled similarity matrix (Fig 5A). Similarly, the contents of the protein-protein similarity matrix were controlled (e.g. half-filled protein similarity, and zero-filled protein similarity) while the full chemical similarity matrix was used. Unlike the chemical-chemical similarity, the protein-protein similarity information did not necessarily improve REMAP’s predictive power. The performance was best when a half of the protein-protein similarity information was used together with the full chemical-chemical similarity matrix (Fig 5B). This suggests that there is significant noise in the protein-protein sequence similarity matrix although the information does help prediction. A careful examination of the BLAST-based protein-protein similarity matrix may give an insight into the design of a novel protein-protein similarity metric for drug-target binding activities (see discussion section).

**Fig 6:**
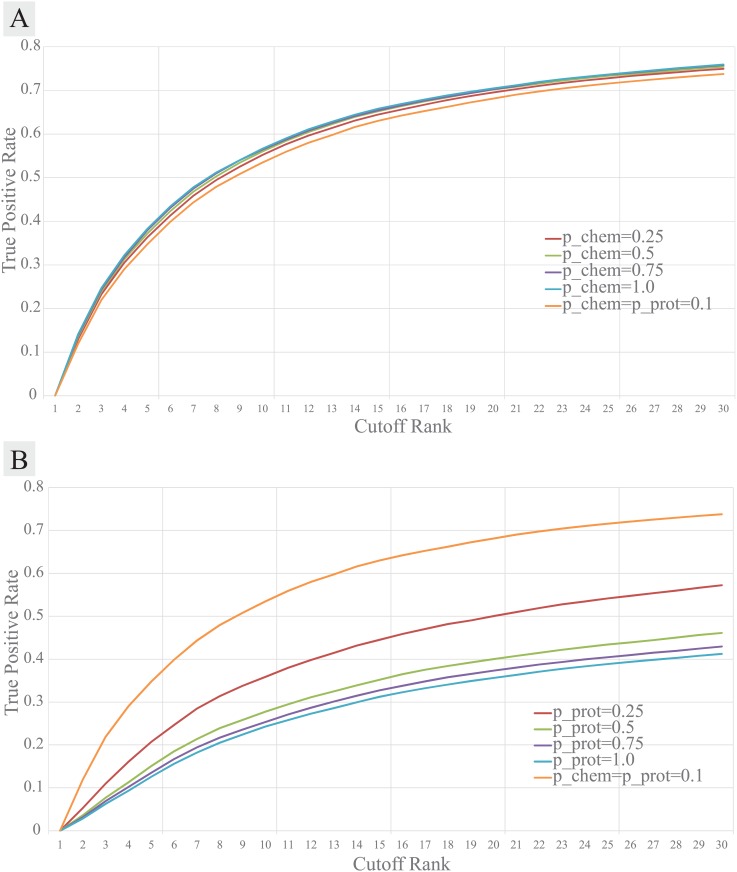
Performance of REMAP according to the importance parameters for the chemical-chemical (*p*_*chem*_) or the protein-protein (*p*_*prot*_) similarity information used for its 10-fold cross validation on the ZINC dataset. **(A)** The chemical-chemical similarity importance parameter, *p*_*chem*_, was controlled while *p*_*prot*_ = 0.1 fixed. **(B)** The protein-protein similarity importance parameter, *p*_*prot*_, was controlled while *p*_*chem*_ = 0.1 fixed.

We also performed optimization tests for *p*_*chem*_ and *p*_*prot*_ on ZINC dataset. Although the performance was slightly better when the chemical-chemical similarity importance was maximum (Fig 6A), the difference was too small to conclude that it is best to fix *p*_*chem*_ = 1. Instead, the prediction may rely too much on the chemical-chemical similarity scores. Therefore, to allow flexibility on chemical-chemical similarity information, we set *p*_*chem*_ = 0.75 at which the performance was almost as accurate as *p*_*chem*_ = 1. On the other hand, the performance was best when the protein-protein sequence similarity importance, *p*_*prot*_, was 0.1 (Fig 6B), further supporting our claim that protein-protein sequence similarity is not an optimal choice for the prediction of a drug-target interaction. When jointly optimizing *p*_*chem*_ and *p*_*prot*_, their optimal value is 0.25 and 0.25, respectively, in the 10-fold cross validation benchmark evaluation (S2B Fig).

Our result supports a recent study [25] which showed that Tanimoto coefficient is efficient for the chemical similarity calculation. Chemical fingerprint-based chemical-protein association prediction has been studied by Koutsoukas et al [6]. By defining bins (target proteins) that can contain certain chemical features based on the chemical fingerprints, Koutsoukas et al. successfully demonstrated that their algorithm, PRW, can efficiently predict unknown chemical-protein associations [6]. While the basic idea of dissecting chemical compounds into functional groups is the same, it should be noted that PRW does not consider the information obtained from proteins, as well as interactome.

### REMAP is readily scalable for large chemical-protein data space

**Fig 7:**
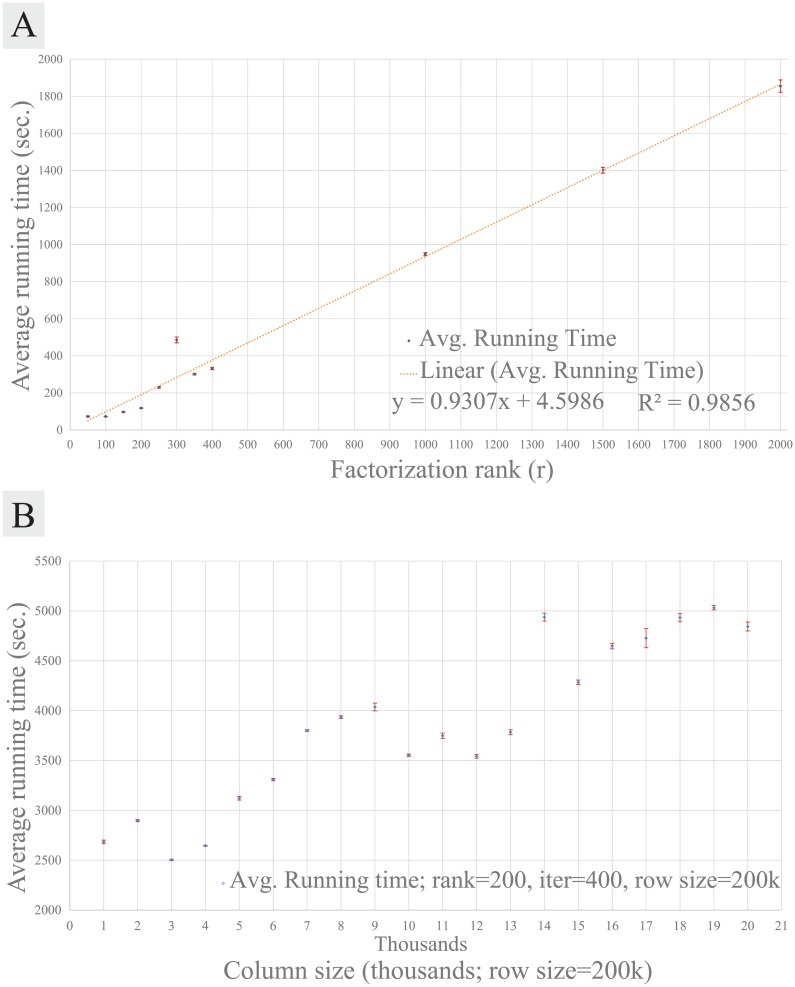
Average running times of REMAP using a single core node with 2.88 GB of memory. All running times are in seconds. **(A)** Average running times on the ZINC dataset (12,384 chemicals and 3,500 proteins) according to the low-rank (*r)*. The linear fit with R^2^ = 0.9856 (orange line). **(B)** Average running times according to the number of proteins (columns) from 1,000 to 20,000. The number of chemicals (rows) were fixed to 200,000. Error bars represent s.e.m., with n ≥ 15 for (A) and n ≥ 30 for (B).

For all our tests, REMAP showed great speed without losing its accuracy. On our benchmark dataset (ZINC; 12,384 chemicals and 3,500 proteins), it took approximately 120 seconds to run 400 iterations at the rank of 200 (*r* = 200, *p*_*iter*_ = 400). The time complexity is linearly dependent on the rank (Fig 7A). The scalability of REMAP is superior when compared to KBMF2K, a state-of the art matrix factorization algorithm that is implemented in Matlab and has been extensively studied for predicting drug-target interactions [16]. KBMF2K took more than 10 days for the same size matrix using the same computer system in the ZINC benchmark. Moreover, REMAP was capable of higher rank factorization while KBMF2K was limited to rank 200 in our system due to the memory requirement (over 100 GB of memory). At a much higher rank (*r* = 2,000), less than one hour was required for REMAP on the same dataset (Fig 7A). Time complexity experiments on larger dataset showed that REMAP completed predictions on a dataset with 200,000 rows and 20,000 columns within 2 hours on a single core computing system with 2.88 GB of memory, demonstrating its ability to screen the whole human genome of approximately 20,000 proteins in two hours (Fig 7B).

### Large scale prediction of drug-target interactions

Since REMAP is scalable and shows superior accuracy based on our benchmark tests, we performed large scale prediction of drug-target interactions on the ZCD dataset (explained in the Materials and Methods section). As explained in the prediction score adjustment section, prediction scores for the active pairs were mostly located between 0.75 and 1.0 (Fig 2A).

### Low rank profile based drug-drug similarity analysis

As expected, the percentage of pairs of chemicals that share common targets decreases with the decrease of the chemical structural similarity measured by the Tc of ECFP fingerprints ($C_{\left(c_{1},c_{2}\right)}$). The percentage of target-sharing chemical pairs drops below 50% and 0.5% when the Tc is between 0.5 and 0.6, and less than 0.5, respectively (S6 Fig). Thus, it is less likely that the chemical structural similarity alone can reliably detect novel binding relations between two chemicals when the Tc is less than 0.5. It is interesting to see how REMAP performs when the chemical structural similarity fails.

We analyzed the low-rank drug profile (matrix *U*_*UP*_) to check whether it represented the target-binding behavior of the drugs. When filtered by low chemical structure similarity ($C_{\left(c_{1},c_{2}\right)}<0.5)$), there are 899,871 drug-drug pairs. Among them, the profile similarity score ($S_{cos,(c_{1},c_{2})}$) of 91,888 pairs is higher than 0.3. With high profile similarity ($0.99\leq S_{cos,(c_{1},c_{2})}\leq 1)$), a total of 1,327 drug-drug pairs were found of which 1,033 pairs shared at least one common known target. S7 Fig shows the percentage of pairs that share the common target in different profile similarity bucket for FDA-approved drugs. This result suggests that REMAP is able to provide a chemical-protein binding profile that cannot be captured by chemical structure similarity alone.

When $S_{cos,(c_{1},c_{2})}\leq 0.3$, the percentage of two drugs that share a common target drops below 50% (S7 Fig). We constructed a drug-drug similarity network by filtering out drug pairs with $S_{cos,(c_{1},c_{2})}\leq 0.3$, then applied the MCL algorithm on the drug-drug network to find clusters of similar drugs. The largest cluster of drugs contained a total of 313 drugs, and their relationships to diseases were examined based on the known associations annotated in CTD [31]. As a result, we found that the drugs are mostly related to mental disorders, including hyperkinesis, dystonia, catalepsy, schizophrenia and basal ganglia diseases as the mostly related diseases. The most frequent known protein targets by the drugs were GPCRs (S1 Table). It is comparable that GPCRs were 1,924 times targeted while kinases were targeted only 55 times. While it is interesting to further examine the cluster, validating all of the possible drug-target pairs in the largest cluster may be inefficient.

**Fig 8:**
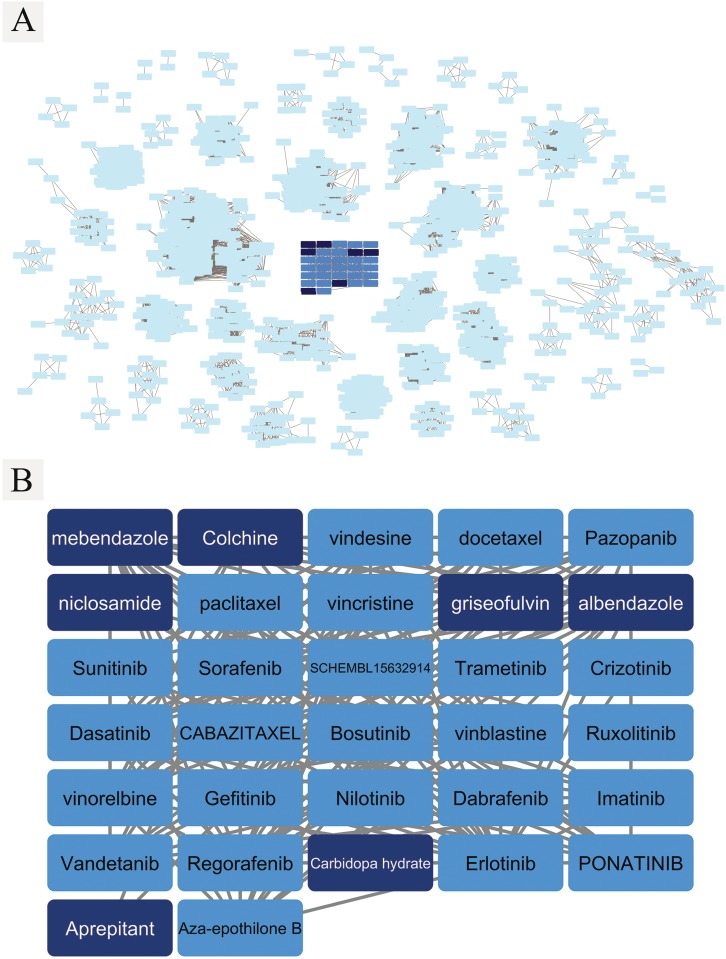
**(A) The drug clusters created based on the profile similarity with the anti-cancer drug cluster in the middle (darker blue grid). (B)** The clusters of FDA-approved anti-cancer drugs. A set of 25 known anti-cancer drugs (blue boxes), and another set of 7 FDA-approved drugs that are closely linked to the former set but have not yet been approved for anti-cancer treatment (darker blue boxes). Procedures explained in the drug-target interaction profile analysis for drug repurposing section.

A smaller cluster of drugs contained a total of thirty-one FDA approved drugs twenty-six of which are known to target kinases or interact with microtubule (Table 2). Seven drugs in the cluster have not been used for cancer treatment and were found to be closely linked to the anti-cancer drugs (Fig 8 and Table 2). Interestingly, several of them have been tested for their anti-cancer activity. For example, colchicine (also known as colchine), an FDA approved drug for gout treatment, has been shown to have anti-proliferative effects on several human liver cancer cell lines at clinically acceptable concentrations [33]. Griseofulvin, an antifungal antibiotic drug, appears to be effective as an anti-cancer drug when used together with other anti-cancer drugs [34]. The three anthelmintic drugs, albendazole, mebendazole and niclosamide, have been studied and repurposed for their anti-cancer effects on different types of cancers. Albendazole has been shown to be effective in suppressing liver cancer cells both *in vitro* and *in vivo* [35], and recently has been repurposed for ovarian cancer treatment with a bovine serum albumin-based nanoparticle drug delivery system [36]. Mebendazole showed anti-cancer activities in human lung cancer cell lines [37] and human adrenocortical cell lines [38], and it has been repurposed for colon cancer treatment [39]. Both niclosamide and mebendazole showed beneficial effects in glioblastoma in different studies [40, 41]. It has been proposed to use aprepitant in combination with other compounds to improve the efficiency of temozolomide, the current standard drug for glioblastoma treatment [42]. Anti-cancer activity of carbidopa hydrate have not yet been reported. It will be interesting to experimentally validate the prediction.

**Table 2:** The known uses and target information for the anti-cancer drug cluster in Fig 8B obtained from DrugBank. The known targets are in UniProt Accession. The target information from UniProt is in S1 Table.

Drug name	Approved treatment(s)	Known binding target(s)	Principal mode of action
Albendazole	Parenchymal neurocysticercosis	F1L7U3, Q71U36, P68371, P83223	Tubulin polymerization inhibitor
Aprepitant	Antiemetic	P25103	Substance P/Neurokinin NK1 receptor antagonist
Carbidopa hydrate	Reduce adverse effects of levodopa in Parkinson disease treatment	P20711	DOPA decarboxylase inhibitor
Colchine	Gout	Q9H4B7, P07437	N/A (depolymerize microtubule)
Griseofulvin	Ringworm infection	P10875, P87066, Q99456	N/A
Mebendazole	Anthelmintic	Q71U36, P68371	Tubulin polymerization inhibitor
Niclosamide	Anthelmintic against tapeworm infections	P40763, O60674, P12931	disrupt oxidative phosphorylation
Aza-epothilone B	Breast cancer	Q13509	Microtubule stabilizer
Bosutinib	Chronic Myelogenous Leukemia	P11274, P00519, P07948, P08631, P12931, P24941, Q02750, P36507, Q9Y2U5, Q13555	Tyrosin kinase inhibitor
Cabazitaxel	Prostate cancer	P68366, Q9H4B7	Microtubule stabilizer
Crizotinib	Non-small cell lung cancer	Q9UM73, P08581	Anaplastic lymphoma kinase inhibitor
Dabrafenib	Metastatic melanoma	P15056, P04049, P57059, Q8NG66, P53667	Inhibitor of some mutant BRAF kinases
Dasatinib	Chronic myeloid leukemia	P00519, P12931, P29317, P06239, P07947, P10721, P09619, P51692, P24684, P06241	BRC/ABL and Src family tyrosine kinase inhibitor
Docetaxel	Breast, ovarian and non-small cell lung cancer	Q9H4B7, P10415, P11137, P27816, P10636, O75469	Microtubule stabilizer
Erlotinib	Non-small cell lung cancer, pancreatic cancer	P00533, O75469	N/A (EGFR inhibitor)
Gefitinib	Non-small cell lung cancer	P00533	EGFR inhibitor
Imatinib	Chronic myelogenous leukemia	A9UF02, P10721, O43519, P04629, P07333, P16234, Q08345, P00519, P09619	Tyrosine kinase inhibitor
Nilotinib	Various leukemias (investigational)	P00519, P10721	Tyrosine kinase inhibitor
Paclitaxel	Lung, ovarian and breast cancers	P10415, Q9H4B7, O75469, P27816, P11137, P10636	Microtubule stabilizer
Pazopanib	Renal cell cancer and soft tissue sarcoma	P17948, P35968, P35916, P16234, P09619, P10721, P22607, Q08881, P05230, Q9UQQ2	Tyrosine kinase inhibitor
Ponatinib	Chronic myeloid leukemia	P00519, P11274, P10721, P07949, Q02763, P36888, P11362, P21802, P22607, P22455, P06239, P12931, P07948, P35968, P16234	Bcr-Abl tyrosine kinase inhibitor
Regorafenib	Metastatic colorectal cancer and gastrointestinal stromal tumors	P07949, P17948, P35968, P35916, P10721, P16234, P09619, P11362, P21802, Q02763, Q16832, P04629, P29317, P04049, P15056, P15759, P42685, P00519	Multiple kinases inhibitor
Ruxolitinib	Myelofibrosis	P23458, O60674	Janus Associated Kinases (JAK) 1 and 2 inhibitor
Sorafenib	Renal cell carcinoma	P15056, P04049, P35916, P35968, P36888, P09619, P10721, P11362, P07949, P17948	Inhibitor of Raf kinase, PDGF, VEGFR 2 and 3
Sunitinib	Renal cell carcinoma and gastrointestinal stromal tumor	P09619, P17948, P10721, P35968, P35916, P36888, P07333, P16234	Multi-targeted receptor tyrosine kinase inhibitor
Trametinib	Metastatic melanoma	Q02750, P36507	Allosteric inhibitor of mitogen-activated extracellular signal regulated kinase 1 and 2
Vandetanib	Broad range tumor types	P15692, P00533, Q13882, Q02763	Inhibitor of VEGFR
Vinblastine	Breast, testicular cancers, lymphomas, neuroblastoma	Q71U36, P07437, Q9UJT1, P23258, Q9UJT0, P05412	N/A (inhibition of mitosis at metaphase)
Vincristine	Acute lymphocytic leukemia, lymphomas, neuroblastoma, rhabdomyosarcoma	P07437, P68366	N/A (inhibition of mitosis at metaphase)
Vindesine	Acute leukemia, malignant lymphoma, Hodgkin’s disease, acute erythraemia, acute panmyelosis	Q9H4B7	Inhibition of mitosis at metaphase
Vinorelbine	Non-small cell lung carcinoma	P07437	N/A (inhibition of mitosis at metaphase)

## Discussion

### REMAP improves the predictive power of off-target prediction and drug repurposing

Our extensive benchmark studies show that REMAP outperforms existing algorithms in most of the cases for the off-target prediction. Compared with other state-of-the-art matrix factorization algorithms, the predictive power of REMAP comes from several improvements. First, we formulated the drug-target prediction as a one-class collaborative filtering problem; thus the negative data are not required for the training. Second, *a priori* knowledge including known negative data can be incorporated into the matrix factorization with imputation and weighting. Finally, using global imputation and weighting, the algorithm is computationally efficient without significantly sacrificing its performance.

The efficiency and effectiveness of REMAP allows us to predict proteome-wide target binding profiles of hundreds of thousands of chemicals. As the proteome-wide target binding profile is more correlated with phenotypic response than a single target binding, REMAP will facilitate linking molecular interactions in the test tube with *in vivo* drug activity. When using a multi-target binding profile predicted by REMAP as the signature of a chemical compound, seven drugs were found to be associated with anti-cancer therapeutics, although they do not have detectable chemical structural similarity. Among them, the anti-cancer activity of six drugs was supported by experimental evidences. Thus, REMAP could be a useful tool for drug repurposing.

### Remaining issues and future directions

Although REMAP showed its high potential on genome-wide off-target predictions as discussed above, two issues remain: the *cold start* problem and suboptimal protein-protein similarity metrics. Similar to matrix factorization algorithms such as NRLMF, REMAP suffers from *cold start* problem, also known as *new user* or *new item* problem. In other words, it is difficult to recommend a product for a *new user* if the *new user* has never purchased or reviewed a product in the database [28]. For novel chemicals that do not have any known target in the dataset, REMAP did not show better performance than PRW. Moreover, if the target of the novel chemical has 5 or fewer known ligands, the recovery of REMAP is lower than 0.5 (S8A Fig). When the novel chemical is similar to those chemicals in the database, the recovery of REMAP reached above 90% (S8B Fig). These results suggest that, in practice, existing matrix factorization-based methods, including REMAP, are not the optimal choice if the chemicals of interest do not have any known target. To resolve this issue, it is possible to design an algorithm that combines the benefits of PRW or other algorithms with REMAP. The use of confidence weights and *a priori* imputation makes it straightforward for REMAP to incorporate additional information. In addition, the time and memory efficiency of REMAP makes it possible to apply active learning to overcome the *cold start* problem [43–46].

The suboptimal performance of REMAP may arise from the lack of molecular-level biochemical details in deriving the protein-protein similarity metrics. When testing the ZINC dataset, we found that REMAP performs better as lower weight was assigned for protein-protein sequence similarity data (Fig 6B). In addition, the predictive power of REMAP improved when about half of the randomly selected protein-protein similarity scores were removed, further confirming that noise confounds relating global sequence similarity to ligand binding (Fig 5B). It is not surprising that proteins with similar sequences do not necessarily bind to similar chemicals, as protein-ligand interaction is governed by the spatial organization of amino acid residues in the protein structure [47]. Amino acid mutations/post-translational modifications and conformational dynamics may alter the binding of the ligand through direct modification of the ligand binding site or allosteric interaction. A protein may also consist of multiple binding sites that accommodate different types of ligands. Thus, two proteins with high sequence similarity do not necessarily bind the same ligands because the two proteins may possess different 3D conformations, especially in their binding pockets [47]. In contrast, two proteins with low sequence similarity can bind to the same ligands if their binding pockets are similar [48, 49]. The binding site similarity can be a more biologically sensitive measure of protein-protein similarity for the off-target prediction [50–55]. Such work is on-going.

### Conclusion

*In silico* drug-target screening is an essential step to reduce costly experimental steps in drug development. In this study, we showed that dual-regularized one-class collaborative filtering algorithm, a class of computational methods frequently used in user-item preference recommendations, may be applied to drug-target association predictions. Our study presents REMAP, a collaborative filtering algorithm with capability of running whole human genome-level predictions within two hours. Other studies on some types of cancer treatment support our algorithm’s ability to capture drug-drug similarities based on both the drug-target interaction profile and the chemical structural similarity. Our study shows the limitation of REMAP in evaluating new chemicals or accommodating biochemical details. Further development of the computational tools for better prediction is needed.

## Supporting Information

S1 Fig True Positive Rate (TPR) determined in 10-fold cross validation of ZINC benchmark set. The e-value cutoffs for protein-protein similarity calculation based on BLAST sequence comparison. The lower the cutoff, the more stringent in similarity detection.
(EPS)

S2 Fig True Positive Rate (TPR) determined in 10-fold cross validation of ZINC benchmark set. (A) parameter selection for rank *r* and the number of maximum iterations. (B) parameter selection for *p*_*chem*_ and *p*_*prot*_.
(EPS)

S3 Fig ROC-like curves comparing the performances of REMAP (green), PRW (blue), and NRLMF (orange) based on the number of known ligands (L). True Positive Rate (TPR) at y-axis vs. cutoff row-rank for the prediction up to the top 10% (350^th^).
(EPS)

S4 Fig ROC-like curves comparing the performances of REMAP (green), PRW (blue), and NRLMF (orange) based on the maximum chemical structural similarity (Tc). True Positive Rate (TPR) at y-axis vs. cutoff row-rank for the prediction up to the top 10% (350^th^).
(EPS)

S5 Fig The performance of NRLMF on the ZINC datasets with varying parameters (r and iter are equivalent to the low-rank parameter, *r*, and the number of iterations, *p*_*iter*_, respectively). The best performance was by r = 100, iter = 300 (yellow bars), which was chosen for the optimized parameters. (A) The optimization on NT1 dataset with varying number of ligands per target. (B) The optimization on NT2 dataset with varying number of ligands per target. (C) The optimization on NT3 dataset with varying number of ligands per target.
(EPS)

S6 Fig The percent of chemical-chemical pairs that share at least one common target in ZINC_ChEMBL_DrugBank dataset. The value for the first bar with the chemical-chemical similarity range between 0.0 and 0.5 is 0.314%.
(EPS)

S7 Fig The percent of FDA-approved drug-drug pairs that share at least one common target in ZINC_ChEMBL_DrugBank dataset. (A) The percent of drug-drug pairs for chemical structure similarity ranges for all FDA-approved drug-drug pairs. (B) The percent of drug-drug pairs for low-rank profile similarity ranges for drug-drug pairs having structure similarity less than 0.5.
(EPS)

S8 FigPerformance comparison for REMAP (green), PRW (blue), and NRLMF (orange).NT1 (1 known target per chemical) datasets used for varying number of ligands (A) and chemical structural similarity (B). Performance measurement explained in the measuring prediction accuracy of REMAP by TPR vs. cutoff rank section. **(A)** Performance comparison on the datasets with varying number of ligands per protein. For example, the x-axis of L1to5 means that the proteins of interest have 1 to 5 known chemicals to bind. **(B)** Performance comparison on the datasets with the ranges of chemical structural similarity of the tested chemicals to the trained chemicals. For instance, the x-axis of Tc0.5to0.6 means that for the tested chemicals, at least one trained chemical was found such that $0.5\leq C_{\left(c_{1},c_{2}\right)}\leq 0.6$ and no trained chemical was found in greater similarity than 0.6. All TPR values are based on 10-fold cross validation. Error bars represents s.e.m. Asterisks represents statistical significance based one t-test of the 10 TPR values (* for p < 0.05, ** for p < 0.001).(EPS)

S1 Table The drugs and target information for the largest cluster in Fig 7. (XLSX)

S2 Table The protein IDs and annotations for Table 2 and S1 Table. Obtained from UniProt. (XLSX)
