# Correction: Large-Scale Off-Target Identification Using Fast and Accurate Dual Regularized One-Class Collaborative Filtering and Its Application to Drug Repurposing

**DOI:** 10.1371/journal.pcbi.1005312

**Published:** 2017-01-03

**Authors:** The PLOS Computational Biology Staff

The affiliation for author Hansaim Lim should read “The Ph.D. Program in Biochemistry, The Graduate Center, The City University of New York, New York, New York, United States” and the affiliation for author Di He should read “The Ph.D. Program in Computer Science, The Graduate Center, The City University of New York, New York, New York, United States”.
